# New insights on hyperglycemia in 17-hydroxylase/17,20-lyase deficiency

**DOI:** 10.3389/fendo.2022.917420

**Published:** 2022-07-22

**Authors:** Lingling Xu, Lin Lu, Anli Tong, Shi Chen, Wei Li, Huabing Zhang, Fan Ping, Yuxiu Li

**Affiliations:** Key Laboratory of Endocrinology of National Health Commission, Department of Endocrinology, Peking Union Medical College Hospital, Chinese Academy of Medical Science, Beijing, China

**Keywords:** 17-hydroxylase/17, 20-lyase deficiency, diabetes, impaired glucose tolerance, high progesterone, hypokelamia

## Abstract

**Objective:**

The adrenal glands of patients with 17-hydroxylase/17,20-lyase deficiency (17OHD) synthesize excessive 11-deoxycorticosterone(DOC) and progesterone, and produce less amount of sex steroid production. Mineralocorticoids and sex hormones play an important role in regulating glucose homeostasis. This study aimed to describe the glucose metabolism in 17OHD patients diagnosed at Peking Union Medical College Hospital (PUMCH).

**Design/methods:**

A total of 69 patients diagnosed with 17OHD after adolescence in PUMCH from 1995 to June in 2021. Among them 23 patients underwent a 3-hours oral glucose tolerance test (3hOGTT) after being diagnosed with 17OHD. Insulin response in patients with normal glucose tolerance (NGT) were further compared between the study two groups with different kalemia status. Another 19 patients were followed up to 30 years and older. All clinical data were obtained from the hospital information system of PUMCH.

**Results:**

*Baseline*: (1) The average body mass index(BMI) of all patients at baseline was 20.3 ± 3.7kg/m^2^. Twenty-three patients underwent 3hOGTT, of whom three were diagnosed with diabetes mellitus, and one with impaired glucose tolerance (IGT). Positive correlation between the ratio of progesterone to upper limit of normal range (P times) and hyperglycaemia was exist(r=0.707, P=0.005). (2) In 19 NGT patients, the insulin concentrations at 0 minute, results of the homeostasis model assessment for β-cell function and insulin resistance were lower in the hypokalaemia group than in the normal kalemia group(7.0(5.8-13.2) *vs* 12.4(8.9-14.9) μIU/ml, P=0.017; 115.5(88.2-240.9) *vs* 253.1(177.2-305.8), P=0.048; 1.54(1.17-2.61) *vs* 2.47(1.91-2.98), P=0.022, respectively). *Follow-up*: Four patients had IGT, while seven patients had diabetes mellitus. Of the 19 patients,11 had hyperglycaemia. P times was significantly higher(7.6(5.0-11.0) *vs* 3.75(2.2-5.3), P=0.008) in hyperglycemia group than in the normal glucose group.

**Conclusions:**

Abnormal glucose metabolism was common in 17OHD patients, which was possibly associated with hypokalaemia and high progesterone levels. Routine monitoring on glucose metabolism in 17OHD patient should be conducted.

## Introduction

Congenital adrenal hyperplasia (CAH) is a group of seven autosomal recessive disorders, each of which involves an enzyme deficiency in the adrenal steroid genesis pathway that leads to the impairment of cortisol biosynthesis (1). More than 95% of congenital adrenal hyperplasia cases are due to 21- hydroxylase deficiency (1) (2). 17-hydroxylase/17,20-lyase deficiency (17OHD) is a rare form of congenital adrenal hyperplasia caused by CYP17A1 gene mutation (3). The CYP17A1 gene encodes an enzyme that expresses both 17-hydroxylase and 17,20-lyase activities in which severe mutations impair the production of adrenal and gonadal sex steroids production and cause sexual infantilism and puberty failure. Both 46, XX and 46, XY patients have female external genitalia and usually present during puberty as girls without secondary sexual characteristics, with hypergonadotropic hypogonadism (4). Corticosterone accumulates and substitutes for cortisol, hence, adrenal crisis rarely occurs in patients with 17OHD. For this reason, children are not usually diagnosed with this condition until they reach adulthood (5). Otherwise, the blockage of the 17-hydroxylase enzyme inhibits cortisol production and consequently increases the production of adrenocorticotropic hormone (ACTH). As a consequence, there is increase accumulation of immediate precursors to the enzymatic block, such as progesterone, mineralocorticoid hormone-deoxycorticosterone (DOC), corticosterone, and progesterone. High concentrations of deoxycorticosterone cause sodium retention, hypertension, and hypokalaemia, with suppression of aldosterone production (6) (7).

Primary aldosternism, another disease caused by excessive secretion of mineralocorticoids, is associated with insulin resistance and hyperglycaemia (8) and hypokalaemia and inhibits insulin secretion (9). However, whether patients with 17OHD, a characteristic of elevated mineralocorticoid hormone DOC levels and hypokalaemia, have insulin dysfunction and abnormal glucose metabolism remains unknown. Therefore, a retrospective study was performed to assess the glucose metabolism in 17OHD patients diagnosed in our hospital.

## Research design and methods

### Patients

This study included 69 patients diagnosed with 17OHD in Peking Union Medical College Hospital (PUMCH) from 1995 to June in 2021. Among them, 23 patients underwent a 3-hours oral glucose tolerance test (3hOGTT) after obtaining a diagnosis of 17OHD prior to medication treatment, while another 19 patients were followed up to 30 years and older. Data on the glucose metabolism in those patients were retrospectively analysed.

A diagnosis of 17OHD was established based on the clinical characteristics and hormone levels. The diagnostic criteria included: hypertension, hypokalaemia, and hypergonadotropic hypogonadism, female patients (46XX) have primary amenorrhea with absent sexual characteristics, whereas 46,XY individuals present with 46,XY DSD with female external genitalia but without uterus and fallopian tubes (3). All clinical data were obtained from the hospital information system of PUMCH. At baseline, all biochemical studies was collected before starting steroids treatment, and the median adrenocorticotropic hormone (ACTH) levels was 137.0(85.1-244.0) pg/ml, cortisol(F) 0.90(0.51-3.15) μg/dl, and oestradiol (E2) levels was 15.6(3.1-27.1) pg/ml (**Table 1**). For patients followed up to 30 years or above, the clinical data was were compared with the findings of either the physical and chemical examinations at the latest follow-up if the glucose metabolism remained normal, or those of examinations at the first time that the abnormal glucose metabolism was recognized.

**Table 1:** Baseline clinical characteristics of 17OHD patients categorized by karyotypes.

N	Total	46XY	46XX	P
	69	46	23	
Age (y)	22.9 ± 8.3	22.4 ± 5.7	23.5 ± 3.7	0.542
BMI (kg/m^2^)	20.3 ± 3.7	20.7 ± 4.2	19.7 ± 2.5	0.245
SBP(mmHg)	158 ± 25	157 ± 24	158 ± 28	0.780
DBP(mmHg)	108 ± 21	108 ± 21	108 ± 22	0.927
K(mmol/L)	3.2 ± 0.6	3.3 ± 0.6	3.0 ± 0.7	0.097
FBG (mmol/L)	5.0 ± 0.9	4.9 ± 0.6	5.2 ± 1.3	0.225
Total cholesterol(mmol/L)	3.8 ± 0.8	3.8 ± 0.8	3.8 ± 0.8	0.911
Triglycerides(mmol/L)	0.9 ± 0.4	0.8 ± 0.4	1.0 ± 0.5	0.074
HDL-C(mmol/L)	1.3 ± 0.4	1.4 ± 0.4	1.2 ± 0.3	0.045
LDL-C(mmol/L)	2.1 ± 0.6	2.0 ± 0.6	2.2 ± 0.6	0.281
Uric acid (μmol/L)	214.9 ± 73.8	225.4 ± 78.4	198.0 ± 64.0	0.196
ALT (U/L)	15.0(11.0-20.0)	16.5(11.0-27.0)	14.0(10.5-30.5)	0.707
Cr (umol/L)	63.8 ± 17.5	66.5 ± 19.8	58.6 ± 11.1	0.104
ACTH(pg/ml)	137.0(85.1-244.0)	163.5(83.4-327.5)	114.0(64.5-267.8)	0.262
E2(pg/ml)	15.6(3.1-27.1)	8.0(0-22.9)	17.4(4.6-23.0)	0.586
P times	7.1(4.7-11.4)	6.9(4.8-11.9)	8.4(6.4-10.7)	0.011

Data was presented as mean(SD) or median(range).

SD, standard deviation; BMI, body mass index; SBP, systolic blood pressure; DBP, diastolic blood pressure; FBG, fasting glucose; HDL-C, high density lipoprotein cholesterol; LDL-C, low density lipoprotein cholesterol; ALT, alanine aminotransferase; Cr, creatinine; ACTH, adrenocorticotropic hormone; E2, estradiol; P times, progesterone concentration/upper limit of normal range.

Ethical approval was not required because the data were properly anonymised and informed consent was only obtained at the time of original data collection. The requirement for obtaining informed consent for this study was waived by the PUMCH ethics board.

### Clinical and laboratory examinations

Physical examinations included age, blood pressure, height, weight. Biochemical examination was performed to determine the renal and liver function, serum potassium concentration, cholesterol (total, low-density lipoprotein, and high-density lipoprotein), triglycerides, ACTH, F, E2, progesterone(P), fasting blood glucose(FBG) and insulin(INS). The normal level for ACTH is <46 pg/ml, and the range for E2 in follicular phase is 22-115pg/ml. Chromosome karyotyping was performed in all patients. In 69 patients with 17OHD, only 23 underwent 3hOGTT prior to the initiation of medication treatment.

### Calculation of variables

1, Body mass index(BMI)=weight(kg)/height squared(m^2^)

2, insulin action was estimated using the homeostasis model assessment for insulin resistance (HOMA-IR).

HOMA-IR = (fasting INS×FBG)/22.5, with glucose and INS expressed as mmol/L and mIU/ml, respectively (10).

3,Insulin secretion was estimated using the homeostasis model assessment for β cell function(HOMA-B).

HOMA-β =20 × fasting INS/(FBG-3.5), with glucose and insulin expressed as mmol/L and mIU/ml, respectively (10).

4, Progesterone times (P times)= progesterone concentration/upper limit of normal women in follicular phase range.

### Statistical analysis

The continuous variables with normal distribution were expressed as the mean ± SD, while the non-normal distribution variables were expressed as the median (interquartile range). The differences in normal continuous variables between groups were detected using the Student t test, non-normal distribution variables were using non-parametric test, and categorical variables were using Fisher’s exact test. Bivariate correlation analysis was conducted between hyperglycemia and clinical data. ANCOVA was used to compare HOMAIR values between the hypokalaemia group and normal kalaemia group in NGT patients, after adjusting for BMI. A P value<0.05 was considered significant in all statistical tests. All analyses were performed using the SPSS version 25.0.

## Result

### Baseline

#### Clinical characteristics of the 17OHD patients

All patients had been assigned female gender at birth and diagnosed with 17OHD after adolescence with confirmed 46, XY (n=46) and 46, XX (n=23) karyotypes. The patients’ average age was 22.9 ± 8.3 years, while the BMI was 20.3 ± 3.7kg/m^2^. No differences were observed in age, BMI values, blood pressure, cholesterol (total and low-density lipoprotein)level, triglyceride level and uric acid level between 46, XY patients and 46, XX patients, however, the progesterone concentration was significantly higher in 46XX patients than in 46, XY patients (P times 8.4(6.4-10.7) *vs* 6.9(4.8-11.9), P=0.011), and HDL-C slightly higher in 46XY patients (1.4 ± 0.4 *vs* 1.2 ± 0.3, P=0.045) (**Table 1**).

#### Insulin action based on the 3hOGTT result

Twenty-three patients underwent 3hOGTT test before medication, of whom three were diagnosed with diabetes mellitus(3/23, 13.0%), one with impaired glucose tolerance (IGT) and 19 patients with normal glucose tolerance (NGT). Average age was 24.4 ± 2.2 years, while BMI was 20.3 ± 0.8kg/m^2^. Four of the 19 patients had hyperglycaemia. Patients with hyperglycaemia were older than NGT, and there were no differences in BMI, blood pressure, lipid profile and uric acid concentration between NGT and hyperglycaemia (**Supplementary Table 1**).

The median HOMA-IR values were 2.3 in 19 NGT patients, and >1.4 in 16 patients with NGT, while 10 had an HOMA-IR value of > 2.0.Thirteen patients suffered hypokalaemia(13/23). Among four patients with hyperglycemia, there were three patients suffering hypokalaemia, the serum potassium concentration were 2.6, 2.9, 1.9mmol/L, respectively. The P times values of four patients with hypgerglycemia were 12.7, 5.98, 4.8, and 10.3, respectively. Bivariate correlation analysis was conducted between hyperglycemia and clinical data, and results showed only a positive correlation between P times and hyperglycaemia(r=0.707, P=0.005).

Patients with NGT were further divided into two groups according to hypokalaemia (< 3.5 mmol/l) status. The INS concentrations at 0 minute were lower in hypokalaemia group compared with that in the normal kalaemia group (7.0(5.8-13.2) *vs* 12.4(8.9-14.9) μIU/ml, P=0.017), but no difference in glucose response was observed between the two groups. Similarly, the HOMA-β value in the hypokalaemia group was lower than in the normal kalaemia group (115.5(88.2-240.9) *vs* 253.1(177.2-305.8), P=0.048). Moreover, the HOMAIR value in hypokalaemia group was lower than that in the normal kalaemia group (1.54(1.17-2.61) *vs* 2.47(1.91-2.98), P=0.022) and such a difference did not disappear after adjusting for BMI (**Table 2**).

**Table 2:** Baseline OGTT results for 19 NGT patients categorized by kalemia situation.

	Hypokalemia (n=11)	Normal kalemia (n=8)	P
0 min BG (mmol/L)	4.7 ± 0.3	4.9 ± 0.5	0.467
30 min BG (mmol/L)	7.5 ± 1.0	7.7 ± 1.2	0.691
60min BG (mmol/L)	6.8 ± 1.2	7.4 ± 1.6	0.377
120min BG (mmol/L)	5.7 ± 1.1	6.0 ± 0.9	0.464
180min BG (mmol/L)	6.0 ± 0.6	5.3 ± 0.8	0.075
0min INS (μIU/ml)	7.0(5.8-13.2)	12.4(8.9-14.9)	0.017
30min INS (μIU/ml)	74.0(55.6-123.4)	126.2(90.4-168.1)	0.055
60min INS (μIU/ml)	53.8(29.8-71.9)	110.3(70.9-115.1)	0.094
120min INS (μIU/ml)	52.2(33.6-68.0)	79.4(48.0-123.0)	0.112
180min INS (μIU/ml)	36.2(24.1-80.8)	39.5(24.1-114.9)	0.849
HOMAIR	1.54(1.17-2.61)	2.47(1.91-2.98)	0.022
HOMAβ	115.5(88.2-240.9)	253.1(177.2-305.8)	0.048

Data was presented as mean (SD) or median (range).

SD, standard deviation; BG, blood glucose; INS, insulin; HOMA-IR, (FINS-fasting glucose)/22.5.

### Follow-up

#### Glucose metabolism

Nineteen patients with NGT have been followed up to over 30 years. Among these patients, four developed IGT, while seven developed diabetes mellitus. One patient who developed diabetes mellitus at the age of 15 years tested negative for genomic markers of maturity-onset diabetes of the young (MODY) and GAD antibody and consequently received metformin; another patient developed IGT at the age of 24 years. Most patients (n=7) developed hyperglycaemia at the age of 30-34 years of age. Of the 19 NGT patients, 11 had hyperglycaemia(58%) (**Table 3**) (**Supplementary Table 2**). The level of progesterone was significantly higher in the abnormal glucose group than in the normal glucose group (P times 7.6(5.0-11.0) *vs* 3.75(2.2-5.3), P=0.008). No significant differences were observed in the karyotypes, lipid profile or uric acid concentration between the two groups.

**Table 3:** The clinical characteristics of 19 patients who followed up to 30 years old or above with NGT at baseline by glucose metabolism status.

	Hyperglycemia(n=11)	Normoglycemia(n=8)	*P*
Age (y)	38.4 ± 11.6	34.8 ± 9.6	0.473
Karyotypes(46XX/46XY)	3/8	2/6	0.481
BMI(kg/m^2^)	20.6 ± 3.3	22.0 ± 5.7	0.506
SBP(mmHg)	163 ± 16	171 ± 25	0.412
DBP(mmHg)	109 ± 13	116 ± 21	0.386
K^+^ (mmol/L)	4.0 ± 0.5	4.0 ± 0.3	0.984
Total cholesterol(mmol/L)	4.2 ± 0.7	4.8 ± 0.6	0.105
Triglycerides(mmol/L)	1.2 ± 0.6	1.4 ± 0.5	0.367
HDL-C(mmol/L)	1.2 ± 0.3	1.4 ± 0.5	0.786
LDL-C(mmol/L)	2.6 ± 0.6	3.0 ± 0.3	0.368
Uric acid (umol/L)	182.0 ± 40.3	224.9 ± 39.4	0.135
ALT (U/L)	25.0 ± 15.0	26.3 ± 13.5	0.862
Cr (umol/L)	61.6 ± 11.7	69.8 ± 10.0	0.136
E2(pg/ml)	9.4(3.2-22.4)	30.6(12.1-49.0)	0.267
P times	7.6(5.0-11.0)	3.75(2.2-5.3)	0.008

Data was presented as mean (SD) or median (range).

SD, standard deviation; BMI, body mass index; SBP, systolic blood pressure; DBP, diastolic blood pressure; HDL-C, high density lipoprotein cholesterol; LDL-C, low density lipoprotein cholesterol; ALT, alanine aminotransferase; Cr, creatinine; ACTH, adrenocorticotropic hormone; E2, estradiol; P times, progesterone concentration/upper limit of normal range.

#### Glucocorticoid and sex hormone treatment

All patients were treated with prednisone or dexamethasone, as a consequence, the patients’ potassium level returned to normal. In NGT group, two patients took prednisone 2.5mg/d, three patients took dexamethasone 0.25mg/d, and other three took dexamethasone 0.375mg/d. In hyperglycemia group, two patients took prednisone 2.5mg/d, one patients took dexamethasone 0.25mg/d, six patients took dexamethasone 0.375mg/d, one took dexamethasone 1.125mg/d, another patients refused to take medication (**Supplementary Table 2**). Thus, there was no difference in two groups. In the normal glucose group (n=8), seven patients received artificial cycle therapy with oestrogen; in the hyperglycaemia group (n=11), only four patients received oestrogen therapy (P=0.057). No significant difference was observed in the serum potassium concentration between the two groups during the follow-up period (P=0.844).

## Discussion

To our knowledge, this study was the first to summarize the baseline and follow-up characteristics of Chinese 17OHD patients. This study summarized the results of physical and chemical examaminations in 69 patients diagnosed with 17OHD at baseline and the glucose metabolism status during follow-up in 19 patients with NGT. The results from this study indicate that impaired insulin secretion is linked with hypokalemia in 17OHD patients with NGT, while hyperglycaemia is associated with high progesterone concentration after hypokalaemia was rectified.

In this study, three of the 23 17OHD patients were diagnosed with diabetes mellitus at baseline, drastically higher than the prevalcence of diabetes among Chinese population aged 18-29 years, the overall standardised prevalence of total diabetes was 2.0%(95% confidence interval 1.5 to 2.7) (11). Abnormal potassium and hormone concentration are involved in the pathophysiologic mechanism of diabetes in these 17OHD patients. The median HOMA-IR value was 2.3 in 19 NGT patients at baseline. Zhang et al. reported 46 normal controls(normal waist circumference + no metabolic abnormality) from Peking Union Medical College physical examination center, among them median HOMA-IR was 0.97(0.66, 1.41) in males, and 1.00(0.81, 1.40) in females (12). HOMA-IR is a convenient and inexpensive surrogate measure of estimating insulin resistance, derived from a mathematical assessment of the balance between hepatic glucose output and insulin secretion from fasting levels of glucose and insulin. HOMA-IR can be used to predict diabetes in the future (13). In the Hong Kong Cardiovascular Risk Factor Prevalence Study (CRISPS)-4, a prospective population-based cohort study with 15 years of follow-up, the optimal HOMA-IR cut-off values of 1.4 and 2.0, were used to distinguish dysglycaemia from NGT, and type 2 diabetes from non-diabetes, respectively (14). In our study, two of three patients had an HOMA-IR values of >1.4, and more than one half had an HOMA-IR value >2.0. Generally speaking, for most 17α-hydroxylase/17,20-lyase deficiency patients, their BMI values are within the normal or lower range due to insufficient cortisol secretion, and they have no other metabolic abnormalities related to insulin resistance, such as hyperlipidaemia and hyperuricemia. In this study, the BMI value, lipid profile, and uric acid level were normal, which indicate that the common risk factor of insulin resistance is hypertension. Thus, the mechanism of insulin dysfunction in 17α-hydroxylase/17,20-lyase deficiency is probably different from that of type 2 diabetes.

Previous literature reported that hypokalaemia also inhibits insulin secretion (15). In this study, no difference was observed in the fasting glucose level, but the INS concentrations at 0 min and HOMA-B value were lower in the hypokalaemia group than in the normal kalaemia group(P=0.017; and P=0.048, respectively). Our results also confirm that hypokalaemia inhibits the islets β Cell function, especially insulin secretion in the basal state. However, HOMA-IR was lower in the hypokalaemia group, even after adjusting for BMI. Therefore, insulin resistance index based on basal insulin secretion may underestimate the degree of insulin resistance in hypokalemia state. The hyperinsulinemic-euglycemic clamp is the gold standard for measuring whole-body insulin resistance (16), thus hyperinsulinemic-euglycemic clamp test is recommended to accurately evaluate the degree of insulin resistance in a hypokalaemia state, especially in patients with 17OHD.

The ACTH-mediated steroidogenesis results of 17OHD patients with elevated concentrations of DOC and corticosterone were obtained. High concentrations of DOC cause sodium retention, hypertension, and hypokalaemia, with suppression of aldosterone production. DOC may inhibit insulin secretion, DOC acetate-induced hypertension was associated with significantly lower levels of plasma insulin in nondiabetic rats (17). Long term glucocorticoid administration will lower the DOC production, but not normalize the DOC concentration(7).Thus, the effect of DOC on insulin secretion may be an important mechanism for hyperglycaemia in 17OHD patients.

Excessive DOC may impair the insulin signaling through oxidative stress and inflammatory mechanism, in DOC acetate(DOCA)+ salt hypertensive rats, oxidative and inflammatory transcriptions factors, such as NF-kB, AP-1, and JNK, which are implicated in insulin resistance are markedly elevated. Moreover, the levels of plasma adiponectin were significantly lower in DOCA+salt hypertensive rats than in controls (18). Unfortunately, DOC was not measuered in the patients in this study.

Sex hormones are important physiological regulators of glucose homeostasis and, of the enteroinsular axis. Oestrogens act on the pancreas to increase the secretion of insulin and glucagon-like peptide-1(GLP-1), and decrease the secretions of glucagon (19). Oestrogen loss during menopause in women is associated with insulin resistance and diabetes (20). Moreover, the lack of oestradiol reduces insulin secretion and increases hepatic insulin degradation (21). Oestrogen replacement therapy can prevent diet-induced ectopic lipid deposition and hepatic and muscle insulin resistance in animal model (22). In the Women’s Health initiative randomized trial, postmenopausal therapy with oestrogen alone may reduce the incidence of treated diabetes (23). In this study, we found that the proportion of patients receiving oestrogen therapy was higher in NGT group than that in the hyperglycaemia group but did not reach statistical significance (P=0.057), indicating that oestrogen may be useful in preventing the development of diabetes in 17OHD patients.

Some diseases or physiological states with elevated progesterone are associated with insulin resistance, such as polycystic ovary syndrome, pregnancy and the luteal phase of the menstrual cycle. Much evidence implicates that progesterone induces insulin resistance. Sex hormones are mostly accumulated and pooled in adipose tissues, the steroid content of adipose tissue is ~10 fold higher than that in the general circulation (24). Wada T et al. found that progesterone inhibits glucose uptake by affecting the PI3-kinase pathway steps of insulin signaling in 3T3-1 adipocytes (25). Otherwise, progesterone receptor was found in pancreatic cells, but uniformly expressed in up to 75% of α-cells in only 5% to 20% of β-cells, suggesting that α-cells might be a more significant target than β-cells (26). Results from this study showed that patients who developed hyperglycemia at 30 years old or over had high progesterone levels than those with normal glucose levels (P times 7.6(5.0-11.0) *vs* 3.75(2.2-5.3), P=0.008), indicating that a high progesterone level may be involved in the diabetes pathogenesis in patients with 17OHD.

Insulin resistance and hyperinsulinemia are also common in 21OHD patients, but the prevalence of hyperglycaemia is not higher in 21OHD patients than in the normal population (27). Both 17OHD and 21OHD patients require long-term glucocorticoid treatment, therefore long-term replacement therapy is not the primary mechanism for hyperglycemia.

Several limitations of this study should be considered. First, OGTT test was not a routine test for 17OHD inpatients, so only some patients underwent OGTT. Second, the lack of DOC measurements suggests that the role of insulin resistance in the development of hyperglycaemia among 17OHD patients remains uncertain. Third, the intensity of insulin resistance was not estimated due to the absence of the hyperinsulinemic-euglycemic clamp test. Fourth, this is a retrospective study with its inherit limitation.

In conclusion, we discovered that abnormal glucose metabolism was common in 17OHD patients, hypokalaemia was linked to insulin secretion, and high progesterone was associated with abnormal glucose metabolism in these patients. All of these findings indicate that hypokalaemia and high progesterone level may contribute to the development of abnormal glucose metabolism. However, further longitudinal investigation with a large sample size and more genetic and biochemical markers are warranted to confirm this causal relationship.

## Data availability statement

The original contributions presented in the study are included in the article/**Supplementary Material**. Further inquiries can be directed to the corresponding author.

## Ethics statement

Ethical review and approval was not required for this study with human participants, in accordance with the local legislation and institutional requirements. Written informed consent was not required for this study, in accordance with the local legislation and institutional requirements.

## Author contributions

LX: data acquisition, analysis, and drafting of the manuscript; LL, AT, CS, WL, HZ, and FP: data acquisition and analysis and interpretation of the data. YL: study concept and design, critical revision of the manuscript for important intellectual content, and study supervision. All authors contributed to the article and approved the submitted version.

## Funding

This work was supported by CAMS Innovation Fund for Medical Sciences(CIFMS)2021-I2M-C&T-B-003.

## Conflict of interest

The authors declare that the research was conducted in the absence of any commercial or financial relationships that could be construed as a potential conflict of interest.

## Publisher’s note

All claims expressed in this article are solely those of the authors and do not necessarily represent those of their affiliated organizations, or those of the publisher, the editors and the reviewers. Any product that may be evaluated in this article, or claim that may be made by its manufacturer, is not guaranteed or endorsed by the publisher.

## Supplementary material

Supplementary Table 1 Baseline clinical data of 23 patients categorized by glycaemia situation. Data was presented as mean(SD) or median(range) SD: standard deviation; BMI: body mass index; SBP: systolic blood pressure; DBP: diastolic blood pressure; HDL-C: high density lipoprotein cholesterol; LDL-C: low density lipoprotein cholesterol.

Supplementary Table 2 Clinical characteristics of the 11 patients with hyperglycaemia followed up to 30 years and older. IGT: impaired glucose tolerance; DM: diabetes mellitus.
