# Supplementary material for: New insights on hyperglycemia in 17-hydroxylase/17,20-lyase deficiency
Source: Front Endocrinol (Lausanne). 2022 Jul 22;13:917420. doi: 10.3389/fendo.2022.917420 (PMC9354396; doi:10.3389/fendo.2022.917420)
Supplement: Supplementary Table 1 — Baseline clinical data of 23 patients categorized by glycaemia situation. Data was presented as mean(SD) or median(range) SD: standard deviation; BMI: body mass index; SBP: systolic blood pressure; DBP: diastolic blood pressure; HDL-C: high density lipoprotein cholesterol; LDL-C: low density lipoprotein cholesterol. [file DataSheet_1.docx]

Supplementary Table 1 Baseline clinical data of 23 patients categorized by glycaemia situation.

|  | NGT | hyperglycaemia | P |
| --- | --- | --- | --- |
| N | 19 | 4 |  |
| Age(y) | 21.6±6.8 | 34.5±8.6 | 0.028 |
| BMI(kg/m^2^) | 20.4±4.2 | 19.5±2.1 | 0.710 |
| SBP(mmHg) | 161.2±20.9 | 156.5±17.7 | 0.682 |
| DBP(mmHg) | 108.1±20.8 | 98.2±9.0 | 0.373 |
| Total cholesterol (mmol/L) | 3.8±0.7 | 3.8±0.5 | 0.951 |
| Triglycerides (mmol/L) | 0.8±0.4 | 1.2±0.3 | 0.257 |
| HDL-C(mmol/L) | 1.3±0.3 | 0.9±0.2 | 0.075 |
| LDL-C(mmol/L) | 2.2±0.6 | 2.5±0.2 | 0.514 |
| Uric acid (μmol/L) | 202.6±72.2 | 247.0±93.3 | 0.431 |

Data was presented as mean(SD) or median(range)

SD: standard deviation; BMI: body mass index; SBP: systolic blood pressure; DBP: diastolic blood pressure; HDL-C: high density lipoprotein cholesterol; LDL-C: low density lipoprotein cholesterol
