# Supplementary material for: New insights on hyperglycemia in 17-hydroxylase/17,20-lyase deficiency
Source: Front Endocrinol (Lausanne). 2022 Jul 22;13:917420. doi: 10.3389/fendo.2022.917420 (PMC9354396; doi:10.3389/fendo.2022.917420)
Supplement: Supplementary Table 2 — Clinical characteristics of the 11 patients with hyperglycaemia followed up to 30 years and older. IGT: impaired glucose tolerance; DM: diabetes mellitus. [file DataSheet_2.docx]

Supplementary Table 2 Clinical characteristics of the 11 patients with hyperglycaemia followed up to 30 years and older.

| No. | karyotypes | Age at first hyperglycaemia recognized  (yr) | Hyperglycaemia state | Latest potassium  (mmol/L) | Hormone treatment after diagnosis |
| --- | --- | --- | --- | --- | --- |
| 1 | 46XX | 31 | IGT | 3.3 | Prednisone 2.5mg qn  artificial cycle hormone replacement |
| 2 | 46XX | 37 | DM | 3.7 | Dexamethasone 0.375mg qd |
| 3 | 46XY | 32 | DM | 4.2 | Dexamethasone 0.375mg qd |
| 4 | 46XY | 34 | IGT | 3.8 | Dexamethasone 0.375mg qd |
| 5 | 46XY | 34 | DM | 4.1 | Dexamethasone 1.125mg qd  Conjugated Estrogens 0.3mg qd |
| 6 | 46XY | 32 | DM | 4.9 | Prednisone 5mg qn |
| 7 | 46XY | 34 | DM | 3.7 | Dexamethasone 0.375mg qd  Estradiol Valerate Tablets 2mg qd |
| 8 | 46XX | 15 | DM | 3.5 | No treatment |
| 9 | 46XY | 33 | IGT | 4.2 | Dexamethasone 0.375mg qd |
| 10 | 46XY | 24 | IGT | 5.2 | Dexamethasone 0.25mg qd  Estradiol Valerate Tablets 2mg qd |
| 11 | 46XY | 39 | DM | 3.9 | Dexamethasone 0.375mg qd |

IGT: impaired glucose tolerance; DM: diabetes mellitus
